# Temporally integrated transcriptome analysis reveals ASFV pathology and host response dynamics

**DOI:** 10.3389/fimmu.2022.995998

**Published:** 2022-12-05

**Authors:** Lin Lv, Tianyun Zhang, Hanying Jia, Yanyan Zhang, Asif Ahsan, Xiaoyang Zhao, Teng Chen, Zhiqiang Shen, Ning Shen

**Affiliations:** ^1^ Department of Infectious Disease, First Affiliated Hospital, College of Medicine, Zhejiang University, Hangzhou, Zhejiang, China; ^2^ Liangzhu Laboratory, Zhejiang University Medical Center, Zhejiang University, Hangzhou, Zhejiang, China; ^3^ Changchun Veterinary Research Institute, Chinese Academy of Agricultural Sciences, Changchun, Jilin, China; ^4^ Shandong Binzhou Academy of Animal Science and Veterinary Medicine, Shandong Academy of Agricultural Sciences, Binzhou, Shandong, China; ^5^ Shandong Lvdu Bio-Sciences and Technology Co., Ltd., Binzhou, Shandong, China; ^6^ Department of Hepatobiliary and Pancreatic Surgery, First Affiliated Hospital, College of Medicine, Zhejiang University, Hangzhou, Zhejiang, China

**Keywords:** ASFV, transcriptome, host-virus interaction, inflammatory, immune response, pathogenicity

## Abstract

African swine fever virus (ASFV) causes a lethal swine hemorrhagic disease and is currently responsible for widespread damage to the pig industry. The pathogenesis of ASFV infection and its interaction with host responses remain poorly understood. In this study, we profiled the temporal viral and host transcriptomes in porcine alveolar macrophages (PAMs) with virulent and attenuated ASFV strains. We identified profound differences in the virus expression programs between SY18 and HuB20, which shed light on the pathogenic functions of several ASFV genes. Through integrated computational analysis and experimental validation, we demonstrated that compared to the virulent SY18 strain, the attenuated HuB20 quickly activates expression of receptors, sensors, regulators, as well as downstream effectors, including cGAS, STAT1/2, IRF9, MX1/2, suggesting rapid induction of a strong antiviral immune response in HuB20. Surprisingly, in addition to the pivotal DNA sensing mechanism mediated by cGAS-STING pathway, infection of the DNA virus ASFV activates genes associated with RNA virus response, with stronger induction by HuB20 infection. Taken together, this study reveals novel insights into the host-virus interaction dynamics, and provides reference for future mechanistic studies of ASFV pathogenicity.

## Introduction

African swine fever, caused by African swine fever virus (ASFV), is a fatal hemorrhagic disease of domestic and wild pigs (1–3). Outbreaks of ASF have spread rapidly throughout Eastern Europe, Africa and Asia, making it a major threat to the pig industry worldwide, especially in the last decade (4, 5). ASFV is one of the most complex DNA viruses known to date, encoding over 150 proteins involved in a variety of stages of ASFV life cycle, including evasion of host immune response, entry into host cells, RNA modification, DNA repair, and virion assembly (6). Macrophages and monocytes are the primary targets of ASFV and are thought to be critical for virus replication and dissemination (6, 7). Despite extensive research on ASFV and its devastating effects on the host, no effective drug or vaccine is available (4). A major restriction in the development of effective ASFV antiviral therapies is due to the limited understanding of the molecular mechanisms of ASFV transcription and its interaction dynamics with the host cell, i.e., studies of a single gene or pathway of ASFV infection fail to provide an integrative understanding of the host-virus interaction dynamics (4, 8, 9). Consequently, comprehensive profiling of ASFV gene expression and its interaction with the host transcriptome is highly valuable, as it may provide novel insights for the development of antiviral therapies and effective vaccines.

RNA sequencing (RNA-Seq) is a high-throughput experiment that can be applied to profile the transcriptome of host and virus during infection (10–13). Using RNA-seq, researchers quantified gene expression levels in Vero cells infected with ASFV-BA71V at early (5 hour) and late (16 hour) stages, providing insights into the temporal expression of known and novel viral genes (14). However, the use of non-ASFV targeted cells is suboptimal and may introduce bias. Some studies also applied RNA-seq to describe the gene expression of porcine alveolar macrophages (PAMs) infected with the highly virulent ASFV strain Malawi LIL20/1, Georgia 2007 or CN/GS/2018, where changes in some important cytokines and transcription factors in host cells after ASFV infection were reported (15–18). The lack of host dynamic transcriptome changes after ASFV infection in this study limited our understanding of continuous biological process within the macrophages. Therefore, a dynamic transcriptome profile of ASFV-target macrophages helps to provide a further view of host cellular response mechanisms to virus.

A great deal of research has been devoted to exploring the pathogenicity of virulent ASFV strain. On the other hand, natural mutated strain showed lower virulence, but high transmissibility, causing chronic and persistent infections in pigs, is less well studied. Previous studies have demonstrated the highly virulent ASFV strains can suppress the immune response while attenuated strains remain unknown in this regard (19–21). One study profiled the blood transcripts of pigs infected with diverse virulence of ASFV strains, indicating distinct gene expression patterns between these strains and associate host genes with macrophages and natural killer (NK) cells, and viral genes with modification of host immunity (15). However, a limitation of this study is that they used mixed cell types, thus transcriptomic changes in the host cells may be complicated by secondary effects in uninfected cells. Another way to study the virulence of ASFV is to compare the pathology of a target-gene deletion with its parental strain. These studies successfully elucidated the functions of the targeted genes and indicated that the genetically modified ASFV strains intend to induce higher levels of immune responses. However, the systematic profile of immune response caused by different virulence of ASFV strain, naturally attenuated strain in particular, remain poorly understood. Thus, elucidation of the host immune response of different virulence strains could provide insightful perspectives on ASFV immune evasion strategies and shed light on new vaccine development strategies.

In this study, we performed RNA-Seq experiments on PAMs infected with virulent (SY18) or naturally attenuated (HuB20) ASFV strains across multiple stages of virus infection, and profiled the transcriptome of the virus and host respectively. Both SY18 and HuB20 strains belong to type II ASFV, where SY18 strain was firstly obtained from specimens in the initial ASF outbreak in northeastern China and caused almost 100% mortality in pigs. HuB20 strain is a naturally attenuated ASFV isolated from southern China that causes 30-40% death in infected pigs, presenting relatively milder clinical symptoms than SY18. We characterized the temporal expression dynamics of both host and virus genes and enriched functions. In particular, we identified differential usage of NF-kappaB and TLRs between SY18 and HuB20, and demonstrated that compared to the virulent SY18 strain, HuB20 activated a rapid induction of strong immune response. Most importantly, we detected genes associated with RNA virus response activated in the infection of DNA virus ASFV, offering new insights into the ASFV pathogenicity. Together, our results provide reference for the host-virus interaction dynamics after ASFV infection.

## Results

### Landscape of host-virus transcriptome dynamics in two ASFV strains

To compare the *in vivo* damages after the infection of ASFV with different virulence, we infected pigs with SY18 and HuB20, respectively. Pigs infected by SY18 developed fever by day 2 after infection and all died within 10 days, while pigs infected by HuB20 had fever by day 12 and died within 21 days (**Figures S1A, B**). In addition, the viral load and pathological damage in blood, lung and heart tissues of HuB20-infected pigs was significantly lower than those SY18-infected pigs (**Figures S1C, D**). Together, the above evidence demonstrates a clear reduction of pathogenicity in pigs infected by HuB20.

**Figure 1:**
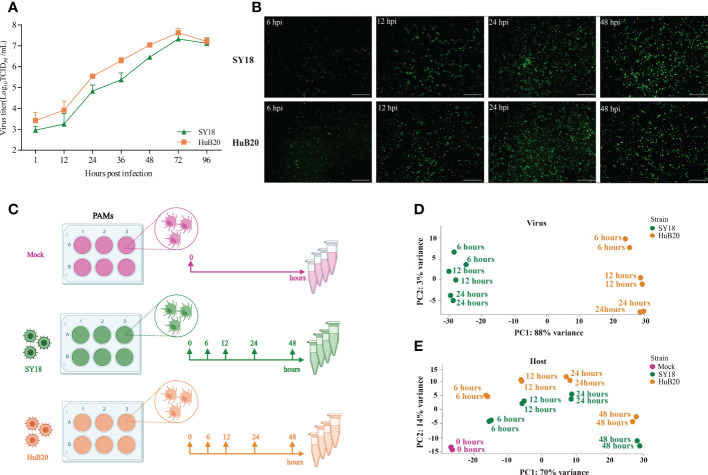
RNA-seq analysis of ASFV strains-infected PAMs. **(A, B)** Identification of ASFV infection. Viral titers in the supernatants were determined by TCID_50_ **(A)**, and immunofluorescence analysis of ASFV viral protein p72 in PAMs infected with ASFV strain SY18 or HuB20 (MOI=0.01) **(B)**. **(C)** The workflow represents the process of sample collection in this study. PAMs were mock-infected or infected with ASFV strain SY18 or HuB20 (MOI= 3), followed by sample collection at 6, 12, 24 and 48 hpi. Total RNA was extracted and polyA enriched RNA sequencing was performed. The principal component of each sample was analyzed considering the virus genes **(D)** or host genes **(E)** expression in the corresponding sample. Samples corresponding to each experimental group were plotted on the first two principal components.

To identify key contributing factors critical for the pathogenicity of ASFV, we profiled the host-virus transcriptome during ASFV infection using RNA-seq, by infecting PAMs with SY18 and HuB20 at 6, 12, 24 and 48 hours post infection (hpi) (**Figure 1C**), respectively. To validate whether PAMs have acquired successful infection, the replication of SY18 and HuB20 viruses was examined by TCID_50_ assay and immunofluorescence assay (IFA) (**Figures 1A, B**). Both viral strains replicated successfully in a time-dependent manner as assessed by the increasing number of ASFV p72 protein-positive cells and the increasing production of infectious viral particles.

Next, we carried out RNA-seq data analysis for samples collected at all time points. Principal component analysis (PCA) of the virus transcriptome suggests that expressional variation between the two virus strains (strain-specific) dominates the transcriptome variation among all samples, explaining 88% of all variation on PC1, whereas time-course expression variation within virus strains only accounts for 3% variation on PC2. This indicates that expressional differences between the virus strains might play a major role in the virulence of the two virus strains (**Figure 1D**). In contrast, transcriptome profiling of the host genome identifies time-course changes as a dominant variation, in contrast to strain specific differences, where 70% of the variance aligned with infection time-course (**Figure 1E**). This indicates that transcriptional responses on the host cells are not distinctly different between the two virus strains, despite distinct clinical outcomes. Nevertheless, we still observed larger variation in the host transcriptome between two virus strains infected with the same timepoint than between biological replicates of the same condition, suggesting that a differential host response in expression exists with infection of the two ASFV strains.

### Temporal analysis of ASFV gene expression programs and functional annotation

**Figure 2:**
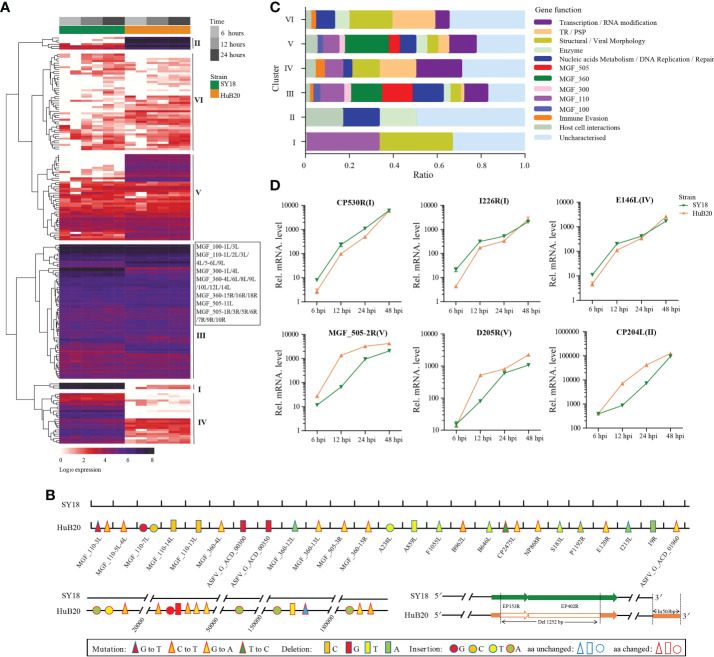
Expression analysis and functional classification of ASFV genes. **(A)** Heatmap shows the expression levels for the 184 viral genes in the ASFV SY18 and HuB20 strains. **(B)** Nucleotide mutations, deletions and insertions in ORFs and the noncoding regions between ASFV SY18 and HuB20 genomes. **(C)** The functional classification of the detected 184 ASFV genes in SY18 and HuB20 strains, annotated with the most enriched function and divided into 6 clusters. **(D)** Validation of randomly selected ASFV gene expression by real-time PCR. At 6, 12, 24, and 48 hours after PAMs were infected with ASFV SY18 and HuB20 strain (MOI= 3), the transcriptional level of CP530R, I226R, E146L (highly expressed in the SY18 strain infected group) and MGF_505-2-R, D205R, CP204L (highly expressed in the HuB20 strain infected group) were detected by RT-qPCR. The fold-difference was measured by the 2^-ΔΔCt^ method. The RNA levels were normalized to the corresponding β-actin.

To study the viral expression programs of SY18 and HuB20, we plotted the temporal gene expression profiles of all viral genes in a replication cycle (6, 12 and 24 hpi), as indicated by the PCA plot of the two virus strain transcriptomes (**Figures S2A, B**). **Figure 2A** demonstrates the viral gene expression profiling of SY18 and HuB20 strains. We identified six clusters according to their expression pattern (**S1 Table**). Cluster III and VI presented similar expression patterns, while cluster I, II, IV and V showed distinct expression programs between the two virus strains. To better understand the difference between these clusters, we annotated the 184 viral genes with functional groups and profiled the functional composition of different clusters (**Figure 2C**). We first looked into the clusters with similar expression pattern. In particular, cluster III contains high expression of MGF 110 family (MGF_110-3L, MGF_110-4L, MGF_110-5-6L), MGF 360 family (MGF_360-12L, MGF_360-13L, MGF_360-15R) and MGF 550-3R at all infection times in both ASFV strains. MGF family genes are extensively distributed in ASFVs and predicted to have large structural and functional diversity. Interestingly, by pairwise genome alignment of these two ASFV strains, we observed multiple mutations, deletions and insertions in HuB20 in these regions (**Figure 2B**). These genomic differences in MGF family may cause reduced pathogenicity in HuB20. Likewise, cluster VI contains genes with high expression in the late stage of infection. Functional annotation for these genes mainly involves structural/viral morphology, transmembrane region/putative signal peptide (TR/PSP), and DNA replication, suggesting that genes associated with viral particle packaging, maturation and propagation were consistently expressed at relatively late timepoints after virus infection for both SY18 and HuB20.

The rest of the clusters encode cluster I and IV, which were expressed higher in SY18, and cluster II and V that were expressed higher in HuB20, respectively. Functional annotations of these gene clusters encompass diverse functional categories, with SY18 high expression cluster IV containing several genes involved in immune evasion, while HuB20 high expression clusters cover more diverse MGF family genes (**Figure 2C**). In addition, we confirmed the differential expression levels of select viral genes using RT-qPCR (**Figure 2D**). It’s also worth mentioning that two large deletions in the 3’ coding region of EP153R and 5’ coding region of EP402R in HuB20 (**Figure 2B**). EP153R was reported to inhibit apoptosis and modulates the expression of MHC class I antigens while EP402R was associated with erythrocyte adsorption. We speculated that the loss-of-function deletions in EP153R and EP402R might be the reason for pathogenic divergence in SY18 and HuB20. Above all, our results suggested distinct differences in viral gene transcription programs between the two strains in one replicated cycle. The dynamic viral gene expression programs and functional annotations in our analysis might contribute to the understanding of the cooperative viral gene functions and pathogenicity differences of ASFV strains with different virulence.

### Host transcriptome dynamics after infection of two ASFV strains

**Figure 3:**
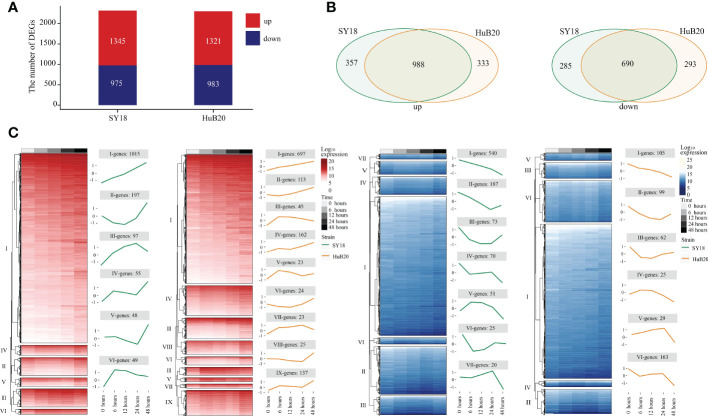
Differentially expressed genes (DEGs) analysis in host with time series. DEGs are examined by the Likelihood Ratio Test (LRT) to explore the genes with significantly differential expression levels across a series of time points (P-value (P) < 10^-10^). **(A)** The stacked plot shows the number of upregulated (red) and downregulated (blue) DEGs of PAMs after being infected by SY18 (left) and HuB20 (right) strains, respectively. **(B)** The Venn diagrams show the shared genes in the two strains for upregulated (left) and downregulated (right) DEGs. **(C)** The heatmap of the DEGs with hierarchical clustering shows the expression levels of upregulated (red) and downregulated (blue) DEGs of PAMs infected by SY18 and HuB20 strains separately. The line plots illustrate the average trend of gene expression in hierarchical clusters.

By analyzing the host transcriptome along different infection stages of the two virus strains, we identified a total of 2320 significant differentially expressed genes (DEGs) (1345 upregulated and 975 downregulated), and 2304 DEGs (1321upregulated and 983 downregulated) in SY18 and HuB20 respectively, compared to mock-infected samples (*P < 10^-10^*) (**Figure 3A**). Meanwhile, approximately two thirds of the DEGs (988 in the upregulated group and 698 in the down-regulated group) were shared by the two strains (**Figure 3B**), confirming that similar level of transcriptome response was stimulated by the two virus strains of different virulence. However, about one third of the DEGs demonstrated specificity between SY18 and HuB20 strains in both upregulated and downregulated group, suggesting that potentially diverse expression programs were involved in the host transcriptome after infection with the two virus strains.

To further investigate the expressional programs in the host response resulting from SY18 and HuB20 infection, we grouped the DEGs into a total of 28 clusters (13 clusters in SY18 strains and 15 clusters in HuB20 strains) of coexpressed genes based on their expression patterns (**Figure 3C**, **S2 Table**). A large fraction of DEGs in the up- and downregulated groups, categorized as cluster I, demonstrated linear expression changes along with time, implying cumulative effects of expressional changes in the host cell after virus infection. Additionally, we observed varying patterns of coexpressed gene clusters across different timepoints, suggesting that multiple dynamic transcriptional programs were involved in the host response.

Next, we sought to identify the transcriptional program regulators, i.e., transcription factors (TFs) enriched in different clusters of coexpressed genes, through MEME motif search on the promoters of the selected genes (**S3 Table**). A number of TFs with known regulatory functions in the immune response, cytokines release, and type I IFN activation were identified, such as SP1, PATZ1, ETV5, STAT2, and IRFs (**Figure S3**). Interestingly, while some TFs, e.g., SP1, ETV5, STATs and IRFs, were enriched in multiple DEG clusters, other TFs showed enrichment in specific clusters. For example, ZN341, a transcriptional activator of STAT1 and STAT3 transcription, whose function was involved in the regulation of immune homeostasis, was upregulated only in cluster II of SY18 host response genes. To sum up, our analysis demonstrates an intricate regulatory network for dynamic host response transcriptional programs.

### Pathway enrichment analysis of host DEGs reveals proinflammatory response after ASFV infection

**Figure 4:**
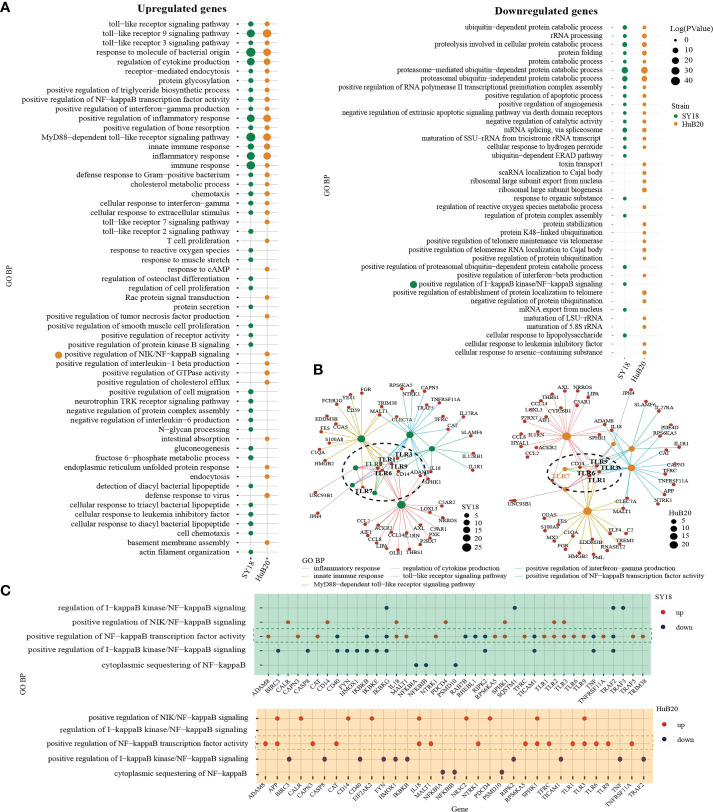
GO analysis of the genes with expression changes at 6, 12, 24, and 48 hpi. **(A)** Gene ontology biological processes (GO-BP) enrichment analysis of upregulated (left) and downregulated (right) DEGs (*P < 10^-10^*) of the two strains separately, and the bubble plot shows the GO terms with *P < 0.01*. **(B)** The network shows the relationship of most enriched up-regulated GO terms in PAMs after being infected by SY18 (left) and HuB20 (right) strains. **(C)** Dot plots of NF-kappaB related GO terms enriched by DEGs of PAMs after being infected by SY18 (top) or HuB20 (bottom) strains.

To understand the pathways and biological processes enriched in the host transcriptome response to ASFV virus infection, we performed Gene Ontology (GO) enrichment analysis for the up- and downregulated (**Figure 4A**, **S4 Table**) DEGs of the two virus strains respectively. As expected, in the upregulated group, DEGs of both strains were significantly enriched in immune and inflammation-associated pathways, including toll-like receptor (TLR) pathway, NF-kappaB transcription activity, cytokines production and interferon gamma production. Interestingly, when we look into the relationship between the genes and top upregulated pathways, we identified TLR genes such as TLR1, TLR3, TLR6 and TLR9 as connector genes amongst different pathways both in SY18 and HuB20 infection (**Figure 4B**), highlighting the upregulation of TLRs as key genes in the host response to ASFV infection. Moreover, we noticed that in addition to TLR9 signaling which primarily recognizes DNA, TLR3 signaling, which is located on the endosome membrane and primarily recognizes dsRNA, was also enriched. This is consistent with previous studies showing ASFV replication in the cytoplasm instead of the nucleus, a unique feature of ASFV compared to other DNA viruses (6, 7, 22). Thus, ASFV replication in the cytoplasm may be responsible for the activation of the TLR3 signaling, which might be a crucial step in inducing the innate immune response and inflammatory responses in the host. Apart from common TLR genes activated in both ASFV strains, we also observed TLR2 specially activated in SY18 while TLR7 only activated in HuB20. Differential usage of these key TLRs and its interaction factors may explain the subsequent diverse host antiviral activities in SY18 and HuB20.

On the other hand, DEGs in the downregulated panel in response to both virus strains were mainly involved in the proteasome-mediated protein catabolic process and apoptotic process, suggesting both ASFV strains were able to inhibit degradation of protein catabolic process and cell death through transcription. Notably, T cell proliferation, defense response to virus, response to cAMP, and positive regulation of interleukin-1 beta production were specifically enriched in the upregulated DEGs of HuB20 infected cells, indicating HuB20 was better at activating the host immune response by stimulating pro-inflammatory cytokine secretion and T cell proliferation. This may be help explain the relatively mild clinical symptoms at the early stage of infection in HuB20. On the other hand, chemotaxis, protein secretion, and negative regulation of interleukin-6 production were enriched only in the upregulated DEGs of SY18 infected cells, indicating the two virus strains might stimulate different cytokines/chemokines response in the host.

To take a deeper dive into the gene-pathway relationships, we next plotted the involvement of all the DEGs related to NF-kappaB signaling in SY18 and HuB20, respectively (**Figure 4C**). NF-kappaB is known as a central pathway in the host cell in response to ASFV infection (23–25). While previous studies reported expression or activity changes in genes related to NF-kappaB, neither a clear picture of the NF-kappaB response to ASFV infection, nor the similarities and differences between strains of different virulence have been described. In our analysis, both SY18 and HuB20 enriched the same NF-kappaB related pathways and regulated gene expression changes in mostly the same directions (**S5 Table**). In particular, genes that are known to activate NF-kappaB, e.g., TLR1, TLR3, TRAF5 and CD14, showed increased expression over infection time. On the contrary, inhibitory genes of NF-kappaB activity, e.g. IKBKB, NFKBIB and IKBKG decreased over infection time (**Figure S4**). Thus, ASFV orchestrated NF-kappaB pathway by regulating the above-mentioned genes expression in host immune and inflammatory response. Despite similar expression pattern in NF-kappaB related genes, we observed differential expression for genes involved in NF-kappaB transcription factor activity (TLR3, IL18, EIF2AK2) (**Figure S4**). These differences might be responsible for the changes of inflammatory factors between SY18 and HuB20. Our analysis reveals, for the first time, the differential expression regulation of NF-kappaB signaling between SY18 and HuB20.

### Diverse cytokines and chemokines responses induced by ASFV infection

**Figure 5:**
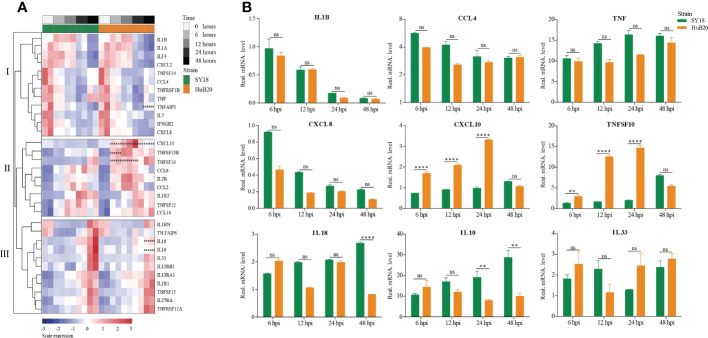
Patterns of cytokines changes and chemokines expression in PAM at different times after ASFV infection. **(A)** Heatmap of cytokines and chemokines expression after ASFV infection. cytokines and chemokines were divided into 3 clusters according to distinct patterns in expression over time. Dots illustrate the significance compared between two strains at the same time point with constraints the absolute value of log_2_foldchange > 1 and *P < 0.1*. *, *P < 0.1*; **, *P < 0.05*; ***, *P < 0.01*, ns, not significant. **(B)** Validation of randomly selected host cytokines and chemokines expression by real time-PCR at 6, 12, 24, and 48 h after PAMs infected by two ASFV strains (MOI= 3). Data are presented as mean ± SD of three independent experiments. The fold-difference was measured by the 2-DDCt method. Differences were assessed using a two-sample t-test. Significance was defined at *P < 0.05* and log_2_foldchange >1. **P < 0.01; ***P < 0.001, ****P < 0.0001, ns, not significant.

Cytokines and chemokines-mediated immune and inflammatory responses are critical for ASFV pathogenicity (26, 27). Despite extensive efforts to study the differences in cytokines and chemokines expression after ASFV infection, results reported thus far remain contradictory (28, 29). To better understand the regulation of cytokines and chemokines by the two ASFV strains over time, we plotted the relative expression profiles of cytokines and chemokines DEGs, and validated their expression by RT-qPCR (**Figures 5A, B**). The cytokines and chemokines DEGs were grouped into three clusters with distinct patterns in expression. The first cluster contains mainly downregulated factors in both SY18 and HuB20 infected cells that are all involved in immune and inflammatory responses. We validated this finding through RT-qPCR, confirming that the expression of the proinflammatory factors IL-1β, CCL4, TNF and CXCL8 decreased progressively with viral infection (**Figure 5B**).

In addition, we noted that the PAMs we used had high initial TNF levels. To investigate their effect on cytokines expression, we used two PAMs with high and low initial TNF expression levels for viral infection, and found that both PAMs had similar cytokines expression trends after infection with ASFV (**Figure S5**).

The second and third cluster cytokines and chemokines genes were all upregulated after virus infection, including interleukins, interleukin receptors, TNF superfamily genes, and C-C motif chemokines. Note that several genes showed significant expression differences between SY18 and HuB20. In particular, CXCL10, TNFSF10, and TNFSF13B, critical regulator or effector genes in immune and inflammatory responses, showed significantly increased expression in HuB20 infection relative to SY18 from 6 hpi, suggesting that these genes might be responsible for the rapid induction of a stronger immune or inflammatory response in attenuated ASFV infection. On the contrary, increased expression of the cytokines IL10 and IL18 at 48 hpi were significantly higher in SY18 compared to HuB20. High levels of IL18 might contribute to the more severe tissue damage caused by the highly virulent strains in later stages of infection. Due to the ability of IL-10 to suppress innate and adaptive responses (30), high levels of this cytokine may favor the pathogenesis of ASFV SY18 by contributing to immune system failure, as reported by other authors (28, 31). Taken together, our cytokines and chemokines analysis revealed integrated and complex regulation of immune and inflammatory responses following ASFV infection. Our analysis suggests differential expression of cytokines and chemokines factors, such as IL10, IL18, CXCL10 and TNFSF10, may be associated with the differential pathogenicity of the two ASFV strains with different virulence.

### Stronger antiviral innate immune response stimulated by HuB20 than SY18

**Figure 6:**
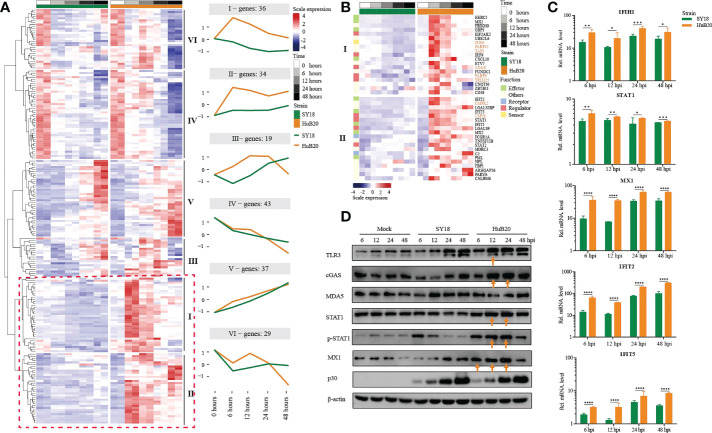
Comparison of host gene expression differences. **(A)** Heatmap of DEGs considering the effects of infection of SY18 and HuB20 separately over time on PAMs with the LRT test. The full model’s design formula includes the effects of infection over time, and the reduced model removes this term to perform the LRT test. The line plots illustrate the average trend of gene expression in clusters. Each cluster has at least 15 genes. **(B)**. Heatmap of the DEGs in clusters I and II, with immune related functions annotated for each gene. **(C)** Validation of innate immunity associated gene expression by real time-PCR. PAMs were infected or mocked infected by ASFV SY18 and HuB20 strains, respectively (MOI= 3), at 6, 12, 24 and 48 hpi. Total RNA was extracted from the PAMs and subjected to RT-qPCR to quantitate IFIH1, STAT1, MX1, IFIT2 and IFIT5 expression. The data were normalized using β-actin. The fold-difference was measured by the 2^-ΔΔCt^ method. Differences were assessed using a two-sample t-test. Significance was defined at *P <0.05*. *, *P < 0.05*, **, *P < 0.01*; ***, *P < 0.001*, ****, *P < 0.0001*. **(D)** Western blotting analysis of innate immunity associated proteins. PAMs were infected or mocked infected by ASFV SY18 and HuB20, respectively (MOI= 3), at 6, 12, 24 and 48 hpi. Cell lysates were collected and subjected to Western blotting analysis using the indicated antibodies.

To further explore the differential expression program in the host response between SY18 and HuB20 along the infection timeline, we considered the interaction term of virus strains and time points and fitted a Likelihood Ratio Test to identify differentially expressed genes. A total of 6 clusters with at least 15 genes in each cluster of similar expression patterns were found (**Figure 6A**, **S6 Table**). Among them, cluster I and cluster II expression patterns differ most. GO enrichment analysis of individual clusters identified cluster I and II were enriched in innate immune response related biological processes such as type I interferon signaling, interferon-stimulated gene 15 (ISG15)-protein conjugation and the JAK-STAT cascade (**S7 Table**). Interestingly, starting from 6 hpi, the innate immune response related effectors (MX1/2, GBP1, HERC5, IFIT2/3/5 and PML) in cluster I and II were early upregulated in HuB20 infected cells while were inhibited or slowly rising in SY18 infected cells (**Figure 6B**), suggesting that the low virulent HuB20 strain could stimulate a rapid immune and defense response in the early stages of infection. However, these immune effectors failed to sustain beyond 24 hpi, indicating the accumulated viral replication may gradually suppress the function of immune related effectors, thereby disturb host immune system.

Apart from immune effectors, we also observed high transcript levels of intracellular sensors and receptors (cGAS (cluster III), *P < 3.82e^-12^*, CD38, *P < 7.85e^-5^* and FCGR1A, *P < 9.96e^-26^*) from 6 hpi of HuB20, whose functions were related to the recognition of viral DNA (32–36). Furthermore, we noted that STAT1, STAT2, IRF9 also exhibited high levels of transcription earlier in HuB20 infection than SY18. Phosphorylated STAT1 and STAT2, together with IRF9 are known to form the interferon-stimulated gene factor 3 (ISGF3) complex, which transcriptionally activates the ISGs (37, 38). In addition, the RT-qPCR results also proved that HuB20 infection induced higher levels of innate immune-related factors in PAMs than SY18 infection (**Figure 6C**, **Figure S5**). We further validated the consistency of transcriptome changes relative to the protein level using Western blotting (**Figure 6D**, **Figure S6**). Albeit delayed activation at protein level compared to transcript level, the innate immune response genes including cGAS, STAT1/pSTAT1 and MX1 all demonstrated high levels of activation in HuB20 infected cells compared to SY18 (**Figure 6D**). Therefore, we suspect that activation of these regulators might contribute to the introduction of a rapid immune response by the attenuated ASFV strain.

Most importantly, we identified up-regulated genes enriched in RNA virus response in HuB20 (IFIH1/MDA5, *P < 1.69e^-16^*, TRIM25, *P < 1.21e^-4^*, USP18, *P < 3.47e^-27^* and PARP9, *P < 1.32e^-2^*). Of note, ASFV infection was considered to be mediated by the DNA-sensing cGAS/STING pathway. The high expression level of these RNA virus response genes in HuB20 enlighten us of a novel biological process activated in ASFV infection. Among these genes, RNA sensor MDA5 recruits the signaling adaptor MAVS to initiate type I interferon signaling and downstream antiviral responses while TRIM25 and USP18 was responsible for regulating antiviral innate immunity by delivering K63-linked polyubiquitin chains to RIG-I (35, 39, 40). Besides, PARP9 was a non-canonical RNA sensor that depends on the PI3K/AKT3 pathway to produce type I IFN. In addition, PARP12, TAP1, CMPK2 and ADAR, which are crucial to restrict RNA virus replication (41–44), e.g. DENV、HIV、ZIKV, also upregulated in the early stages of HuB20 infection, indicating multiple antiviral responses was involved in this stage. In general, our findings presented infection of the DNA virus ASFV activates genes involved in RNA virus response, offering novel mechanistic insights in ASFV pathogenicity.

### Host-virus coexpression network reveals new insights into the functions of viral genes

**Figure 7:**
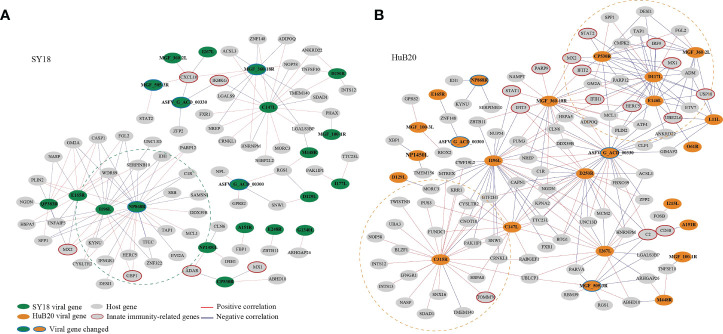
The correlation between the ASFV and host DEGs was measured using Pearson correlation coefficients for the corresponding gene expression and is visualized in the network for SY18 **(A)** and HuB20 **(B)** strains. The absolute value of the Pearson correlation coefficient > 0.9 was considered significant. Red and blue lines indicate positive and negative correlations, respectively.

To explore the relationship between host and virus gene expression dynamics, we constructed a host-virus coexpression network for each of the ASFV strains (**Figure 7**, **S8 Table**). First of all, we observed a module in the SY18 network with multiple viral genes sharing similar connection to a set of host genes (**Figure 7A**). In particular, I196L as a hub gene shared 88% (15/17) of host coexpression with NP868R, suggesting these two viral genes might be coregulated or involved in similar interactive processes with host cells.

Secondly, we noticed that in the HuB20 network (**Figure 7B**), a module involving MGF_360-2L, CP530R, E146L and D117L viral genes was negatively correlated with innate immunity-related genes, e.g. IRF9, USP18, UBE2L6, IRF9, STAT2, MX1/2, IFIT2 and HERC5. Among these viral genes, MGF_360-2L has been shown to be involved in the pathogenicity of ASFV in pigs, where deletion of multiple MGF360 family genes increased the expression of ISG and type I IFNs in infected macrophages (45–47). However, CP530R, E146L and D117L have never been reported to be associated with innate immunity, but the expression levels of these genes in HuB20 infected cells was all significantly lower compared to SY18, especially at the early stage (**Figure S7**). Thus, we reasoned that the low expression of the three viral genes might play a role in the early antiviral response of host cells to HuB20. In addition, RT-PCR results demonstrated that the expression level CP530R and E146L in HuB20 caught up with SY18 at 48 hpi, this may explain the suppressed immune activity in the late stage of HuB20 infection. Further investigation into these viral genes would help to elucidate their functions in ASFV pathogenicity.

In addition, the viral gene C315R, which encodes TFIIB-like transcription factor, is involved in the regulation of RNA transcription and modification (48). Indeed, we identified positive correlation of C315R with RNA polymerase I subunit F (TWISTNB), integrator complex subunit 12 (INTS12) and mtr4 exosome RNA helicase (MTREX) transcription, which were involved in RNA processing and splicing. Intriguingly, C315R was also associated with genes involved in protein transport between the endoplasmic reticulum (ER) and Golgi, such as NOP58 ribonucleoprotein (NOP58), basic leucine zipper nuclear factor 1 (BLZF1), sorting nexin 16 (SNX16), SDA1 domain containing 1 (SDAD1) and nuclear autoantigenic sperm protein (NASP), indicating possible functional association of C315R with protein transport of the host cells.

Lastly, the same viral genes (e.g. MGF_360-18R, I196L and C147L) often associate with different host genes between SY18 and HuB20 infected cells, suggesting a highly dynamic interactive relationship between the virus and host expression programs. Therefore, elaborating the transcriptional correlation between host and virus genes might provide novel insights to explore the unknown functions of some viral genes, or provide a reference map for target selection to guide vaccine or drug development for ASF disease.

## Discussion

RNA sequencing (RNA-seq) has been applied to study various biological processes, such as revealing the interaction of virus infection and host response (10, 13, 49). However, studies using RNA-seq to preform transcriptomic profiling of ASFV and infected host cells are scarce, and these studies only target a single strain or time point and do not provide a comprehensive picture of host-virus interactions (8, 14–17). Here, we integrated RNA-seq analysis to examine and compare the transcriptomic landscape of porcine PAMs during infection with virulent (SY18) and attenuated (HuB20) ASFV strains at different stages of infection, depicting unprecedented details about the temporal host response after ASFV infection. Regulation of host immune response by ASFV and its mechanisms in pathogenicity are key factors to guide vaccine development. By combining functional enrichment analysis and experimental validation, we highlight similarities and differences in viral and host gene expression patterns and host immune response. In particular, we elucidated differences in host innate immune and inflammatory responses stimulated by two ASFV strains, clarifying a stronger antiviral response early activated in attenuated ASFV strain. In addition, we detected genes associated with RNA virus response activated in the infection of DNA virus ASFV, which may provide novel insights for intensive study of ASFV pathogenicity and therapeutic targets.

Our genome and transcriptome analysis of virus genes depicted distinct profiles of two ASFV strains. Virulence related genes, e.g. I226R, A238L and I177L, were differentially expressed in SY18 and HuB20, implying an important role of pathogenicity in these viral genes. For example, target deletion of I226R in SY18 has been proven to elicit robust immunity in pigs to ASFV (50). Moreover, host-virus interaction helped us to characterize some viral genes of unknown function. Of these viral genes, CP530R, E146L and D117L were negatively correlated with host innate immunity-related genes, indicating they may have potential immunosuppressive function.

Several transcriptomic and other experimental studies have shown that ASFV infection leads to changes in the transcription of pattern recognition receptors (PRRs) in some TLR signaling pathways, as well as significant changes in the transcription of some antiviral and inflammatory factors (21, 51–53). In addition, recently published work profiled transcriptomes of swine macrophages infected with ASFV through single-cell RNA sequencing and was also exhibited the activation of antiviral signaling pathways with increased expression levels of interferon-stimulated genes and inflammatory- and cytokine-related genes (54). Therefore, clarification of the specific inflammatory and immune responses after ASFV infection is essential for resolving the host antiviral response. Our data show that the upregulation of multiple TLRs (e.g. TLR1, 3, 6,9) acts as connectors mediating the regulation of multiple responses, especially cytokines and chemokines production and innate immune signaling (**Figure 4B**). In addition, TLR2 specially activated in SY18 while TLR7 only activated in HuB20. Previous studies reported TLR2 is responsible for the inflammatory IL-6 response, whereas TLR7 signaling is crucial for production of IFNα. Differential activation of TLRs and downstream inflammatory factors between SY18 and HuB20 may explain the subsequent diverse host antiviral activities.

Furthermore, previous literature has shown that infection with ASFV of different virulence can lead to differential inflammatory responses, immune responses and apoptotic processes, while the relevant mechanisms remain unclear. Interestingly, in our results, we noticed that DEGs in both SY18 and HuB20 infection were enriched to the NF-kappaB signaling pathway. By comparing the unique DEGs involved in NIK/NF-kappaB signaling and predicting their enrichment in other known pathways (**Figure 4C**, **S6 Table**), we found substantial similarities and differences between SY18 and HuB20, which may account for the different host responses they elicited. The above results provide new insights and research targets into the role of NF-kappaB-regulated immune, inflammatory and apoptotic responses in ASFV infection. Certainly, further experimental data to confirm these observed relationships will facilitate the study of the mechanisms by which ASFV regulates host responses.

Our study was limited by a single viral infection dose, within sample cell heterogeneity, individual gene variability and other confounding factors, such as annotation of the reference genome. However, we compensated for the differences caused by individual cell heterogeneity to some extent by comparing and analyzing the gene expression patterns of the two different virulent ASFV strains over time as well as the overall regulatory pathway changes. Meanwhile, combined with previous studies, we analyzed and presented predictive results for a comprehensive set of regulatory pathways and persuasive targets of action following ASFV infection, which will provide insightful information for further investigations to understand the host response after ASFV infection and valid information for screening candidate targets for ASFV inhibition. Future ASFV related genomic datasets could provide the research community with important resources for ASFV studies.

## Materials and methods

### Cells, viruses and antibodies

Porcine alveolar macrophages (PAMs) were prepared from 2-month-old piglets bronchoalveolar lavage as described previously, cultured in Roswell Park Memorial Institute (RPMI) 1640 medium (Gibco, USA), supplemented with 10% fetal bovine serum (Gibco, USA) and grown at 37°C under 5% CO_2_ atmosphere. The ASFV SY18 strain (GenBank accession no.MH766894), a field virulent ASFV, was originally isolated from specimens in the initial ASF outbreak in China (55). The ASFV HuB20 strain (GenBank accession no.MW521382), a natural attenuated ASFV was isolated from the tissues of pigs in Hubei, China (56). The two viruses were passaged in primary PAMs and maintained at -80°C in the biosecurity level 3 lab. The monoclonal antibodies for cGAS, TLR3, STAT1 and p-STAT1 were purchased from Santa Cruz Biotechnology, USA, and anti-β-actin, IFIH1/MDA5 and MX1 were purchased from Proteintech Biotechnology, USA.

### Sample preparation for RNA-sequencing

PAMs (10^6^ per well) were seeded in 6-well plates and mock-infected or infected with indicated ASFV strains at a multiplicity of infection of 3. After 1 hour of incubation, the viruses were removed, the cells were washed twice with PBS, and fresh 1640 medium was added. At the specified time points (0, 6, 12, 24 and 48 hpi), cells were harvested for RNA extraction.

### Library preparation and RNA sequencing

The cDNA libraries were prepared according to the standard Illumina protocol (NEBNext^®^ Ultra™ II RNA Library Prep Kit for Illumina^®^). Briefly, total RNA from the specified PAMs was treated with RNase-free DNase I (Vazyme, China) following the manufacturer’s instructions. The total amount and integrity of RNA was estimated using the Bioanalyzer 2100 system (Agilent Technologies, USA) with the RNA Nano 6000 Assay Kit. First strand cDNA was synthesized using random hexamer primers and M-MuLV Reverse Transcriptase, and then RNaseH was used to degrade the RNA. Second strand cDNA synthesis was subsequently performed using DNA Polymerase I and dNTPs. After commencing PCR amplification, the PCR product was purified by AMPure XP beads, and the library was finally obtained. The libraries were quantified by an Agilent 2100 bioanalyzer and then subjected to sequencing using an Illumina NovaSeq 6000 sequencer.

### Cell total RNA isolation and real−time quantitative PCR (RT-qPCR)

PAMs in 6-well plates were infected with ASFV SY18 and HuB20 at 3 MOI (multiplicity of infection), respectively. Mock-infected cells were used as control. Cells were collected at 6, 12, 24 and 48 hours post inoculation, and total RNA was isolated using a Total RNA Kit I (Omega Bio-Tek, USA) and reverse transcribed with HiScript II Q RT SuperMix (Vazyme, China) according to the manufacturer’s instructions. qPCR was performed using ChamQ Universal SYBR qPCR Master Mix (Vazyme, China) on a LightCycler^®^ 480 Real-Time PCR System. The relative expression of mRNA was calculated based on the comparative cycle threshold (2^^-ΔΔCt^) method and normalized with porcine β-actin mRNA levels. The primer sequence information is provided in **S9 Table**. The results were analyzed using GraphPad Prism 6 software.

### Western blotting analysis

Cell lysates were subjected to 10% SDS-PAGE and then transferred to nitrocellulose (NC) membranes. The membranes were blocked for 1.5 h at room temperature in Tris-buffered saline containing 10% nonfat dry milk and 0.05% Tween 20 (1×TBST) and were incubated at 4°C for 12 h with the indicated antibodies. The Membranes were washed with 1×TBST, incubated with horseradish peroxidase (HRP)-labeled goat anti-rabbit IgG or anti-mouse IgG antibody (Beyotime, China) at room temperature for 45 min, and treated with enhanced chemiluminescence (ECL) reagent (Thermo Fisher Scientific, USA).

### RNA-seq data quality control and read mapping

Raw data (sequencing reads) in fastq format were first processed through in-house Perl scripts. In this step, quality control followed by data cleaning (clean reads) were performed by removing reads containing adapters, reads containing N bases and low-quality reads from the raw data. All downstream analyses were based on cleaned data (v0.20.1) (57). The clean reads were aligned to the Sus Scrofa Largewhite_V1 (GCA_001700135.1) and ASFV (SY18 (GenBank accession no.MH766894) and HuB20 (GenBank accession no.MW521382)) genome assemblies using STAR (v2.7.8a) with default parameters (58). Uniquely mapped read pairs were counted using featureCounts (v2.0.1) (59). To make the count matrix more comparable among samples, normalization is the critical process of scaling raw counts. Hence, the count matrix was normalized based on the median of ratios method using the R package DESeq2 (v1.32.0), and the rlog transformation was performed for PCA plots (60).

### Differential expression analysis

Differential expression analysis was performed using DESeq2 exploiting the likelihood ratio test (LRT) and testing a full and reduced formula for time-course analyses for each strain of the virus separately with a *P < 10^-10^* (**Figure 3**). LRT is used to explore whether there are significant differences in the effect of the timeline. However, for those differentially expression genes, the expression variations were not consistent. Hence, after filtering genes that were significantly different over time, we clustered the genes using DEGreport (v1.28.0) in R to sort genes into groups based on shared expression patterns (61). In addition, we compared the gene expression levels of PAM cells infected by two strains at each time point to explore significant genes with padj < 0.1 and the absolute value of log_2_foldchange > 1 by DESeq2 Wald Test (**Figure 5**).

We were also interested in the differences in gene expression between SY18 and HuB20 infection over time. In other words, we wanted to compare expression levels by considering two conditions, virus and time, at the same time. Hence, we used a design formula for our full model to explore the difference between the two strains in the effect of the infection over time (virus: time). To perform the LRT test as before, we also need a reduced model without the interaction term virus: time. After applying the LRT test, significant genes were identified using a threshold padj < 0.01 (**Figure 6**). Differentially expressed viral genes were identified using a similar method, and the Pearson correlation coefficient between the host significant gene and the viral significant gene was computed.

Further analysis of ASFV genes used their noted or predicted functions, from the VOCS tool database (https://4virology.net/) entries for the SY18 and HuB20 genomes.

### Enrichment analysis of differentially expressed genes

Gene ontology (GO) analysis was performed on the differentially expressed genes on the DAVID 2021 website with default parameters using direct GO biological process categorization (62). Then we displayed the most significantly enriched terms (*P < 0.01*) in bubble plots. However, to better understand the potential biological complexities in which one gene may involve multiple categories, we used the tool provided by clusterProfiler (v4.0.5) in R to depict the linkage of genes and GO terms as a network (63).

### Promoter motif analysis

Two hundred bases upstream and fifty bases downstream of the transcriptional start site (TSS) sequences of genes in each cluster (13 clusters in SY18 and 15 clusters in HuB20) were extracted from the large white genome (GCA_001700135.1) in FASTA format using BEDtools. Sequences were analyzed in MEME (http://meme-suite.org) by using Human DNA motif database (HOCOMOCOv11 core HUMAN mono meme format) for enrichment. The output from MEME is a list of the sequences for which the E-value is less than 10. For the site positional distribution diagrams, all sequences were aligned on their centers. Position frequency matrices (PFMs) of motifs of interest were drawn by the R package motifStack (v1.40.0) (61).

## Data availability statement

The datasets presented in this study can be found in online repositories. The names of the repository/repositories and accession number(s) can be found below: https://ngdc.cncb.ac.cn/gsa/, PRJCA009503.

## Ethics statement

The animal experiment was approved by the Animal Welfare and Ethics Committee of Changchun Veterinary Research Institute under IACUC approval number AMMS-11-2020-018.

## Author contributions

NS conceived the study. NS, ZS, TC, and LL designed the experiment. LL and YZ performed the experiments. TZ, AA, and XZ analyzed the data. NS, ZS, and TC directed the study. NS, LL, HJ, and TZ wrote the paper. All authors contributed to the article and approved the submitted version.

## Funding

This work is funded by the Starting Fund from Zhejiang University and Shandong Major Project of the Science and Technology Innovation (2019JZZY020606).

## Acknowledgments

Thanks for the technical support by the core facilities of Zhejiang University Medical Center and Liangzhu Laboratory.

## Conflict of interest

Author ZS is employed by Shandong Lvdu Bio-Sciences.

The remaining authors declare that the research was conducted in the absence of any commercial or financial relationships that could be construed as a potential conflict of interest.

## Publisher’s note

All claims expressed in this article are solely those of the authors and do not necessarily represent those of their affiliated organizations, or those of the publisher, the editors and the reviewers. Any product that may be evaluated in this article, or claim that may be made by its manufacturer, is not guaranteed or endorsed by the publisher.

## Supplementary material

Supplementary Figure 1 Pathogenicity of ASFV SY18 and HuB20 strain in piglets. **(A)** Daily rectal temperatures of all the piglets after challenge. **(B)** Survival curves of piglets after challenge with indicated strains. **(C)** Quantification of viral DNA loads in selected tissues with real-time PCR. Viral DNA copy numbers were determined with primers specific for p72. **(D)** Histological analysis of lungs, spleen, thymus and inguinal lymph nodes from pigs after challenge with SY18 and HuB20 strain. (hematoxylin and eosin staining, 100× magnification).

Supplementary Figure 2 PCA analysis of the virus. Principal component analysis of SY18 strain **(A)** and HuB20 strain **(B)** from 6 hours to 48 hours after infection.

Supplementary Figure 3 Promoter motif analysis. Promoter motif analysis identifies significantenrichment of the IRF motif (i.e., the IRF1 binding site) and the othermotifs in each cluster (13 clusters in SY18 and 15 clusters in HuB20). Bydefault, two hundred bases upstream and fifty bases downstream of thetranscriptional start sites (TSS) sequences were analyzed in MEME (Evalue<10).

Supplementary Figure 4 Expression differences of NF-kappaB pathway associated genes. **(A)** Heatmap showing expression levels for the NF-kappaB pathway associated genes in the ASFV SY18 and HuB20 strain.

Supplementary Figure 5 Validation of cytokines expression by real time-PCR. PAMs were infected or mocked infected by ASFV SY18 and HuB20 strains, respectively (MOI= 1), at 6, 12, 24 and 48 hpi. Total RNA was extracted from the PAMs and subjected to RT-qPCR to quantitate TNF, CXCL10, TNSF10.IL-1β, IFIT2 and STAT1 expression (A). “High” represents PAMs with high initial TNF expression and "Low"represents PAMs with low initial TNF expression. The data were normalized using β-actin. The fold-difference was measured by the 2-∆∆Ct method.Differences were assessed using a two-sample t-test. Significance was defined at P < 0.05. *, P < 0.05, **, P < 0.01; ***, P < 0.001, ****, P < 0.0001,ns, not significant.

Supplementary Figure 6 Validation of innate immunity associated gene expression by real time-PCR. PAMs were infected or mocked infected by ASFV SY18 and HuB20 strains, respectively (MOI= 1), at 6, 12, 24 and 48 hpi. Total RNA was extracted from the PAMs and subjected to RT-qPCR to quantitate IFIH1, STAT1, MX1, IFIT2 and IFIT5 expression. The data were normalized using β-actin. The fold-difference was measured by the 2-∆∆Ct method. Differences were assessed using a two-sample t-test. Significance was defined at P < 0.05. *, P < 0.05, **, P < 0.01; ***, P <0.001, ****, P < 0.0001, ns, not significant.

Supplementary Figure 7 Western blotting analysis of innate immunity associated proteins. Insertedvalues indicated relative proteins expression in comparison with b-actin.

Supplementary Figure 8 Expression differences of selected ASFV genes. **(A)** Heatmap showingexpression levels for the selected virus gene CP530R, E146L, D117L andMGF_360-2L in the ASFV SY18 and HuB20 strain.

Supplementary Table 1 ASFV gene counts and functional annotation.

Supplementary Table 2 Host gene counts and DEGs with time series.

Supplementary Table 3 Host gene promoter motif.

Supplementary Table 4 GO BP enrichment of host DEGs with time series.

Supplementary Table 5 Host DEGs involved in NF-kB related pathways.

Supplementary Table 6 Classification of DEGs between two strains across time points.

Supplementary Table 7 GO BP enrichment of DEGs in clusters I and II between the two strainsacross time points.

Supplementary Table 8 Pearson correlation between viral DEGs and host DEGs.

Supplementary Table 9 Primers for real time-PCR validation.
