# Supplementary figures and images for: Temporally integrated transcriptome analysis reveals ASFV pathology and host response dynamics
Source: Front Immunol. 2022 Dec 5;13:995998. doi: 10.3389/fimmu.2022.995998 (PMC9761332; doi:10.3389/fimmu.2022.995998)

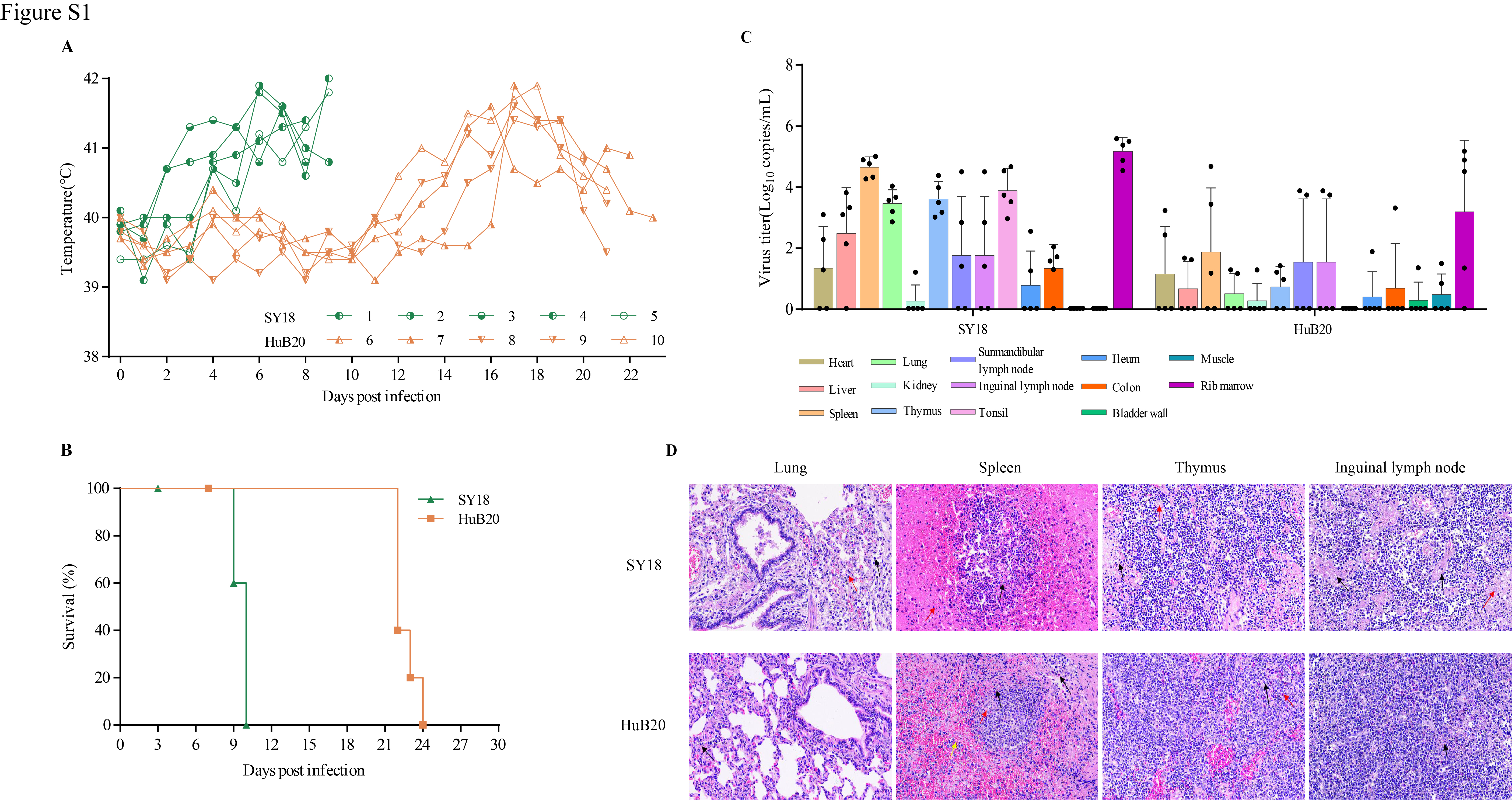

Supplement: Supplementary Figure 1 — Pathogenicity of ASFV SY18 and HuB20 strain in piglets. (A) Daily rectal temperatures of all the piglets after challenge. (B) Survival curves of piglets after challenge with indicated strains. (C) Quantification of viral DNA loads in selected tissues with real-time PCR. Viral DNA copy numbers were determined with primers specific for p72. (D) Histological analysis of lungs, spleen, thymus and inguinal lymph nodes from pigs after challenge with SY18 and HuB20 strain. (hematoxylin and eosin staining, 100× magnification). [file Image_1.jpg]

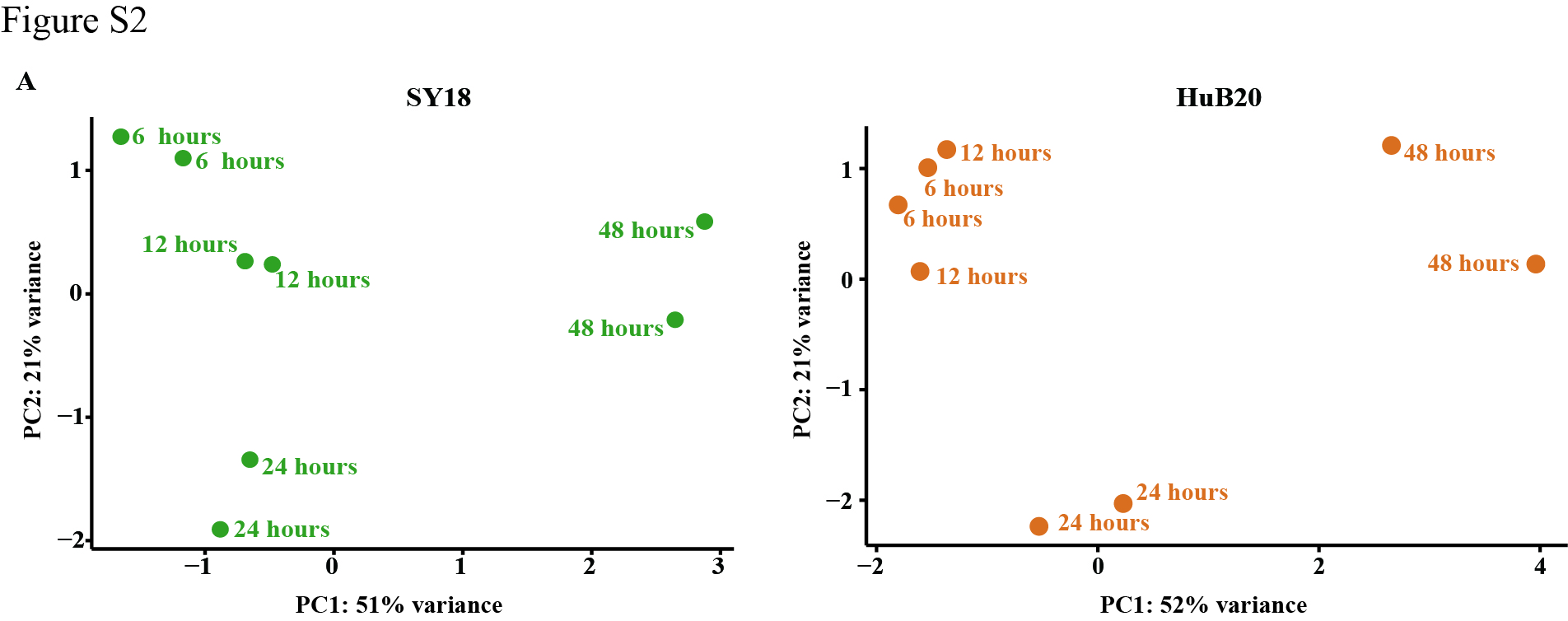

Supplement: Supplementary Figure 2 — PCA analysis of the virus. Principal component analysis of SY18 strain (A) and HuB20 strain (B) from 6 hours to 48 hours after infection. [file Image_2.jpg]

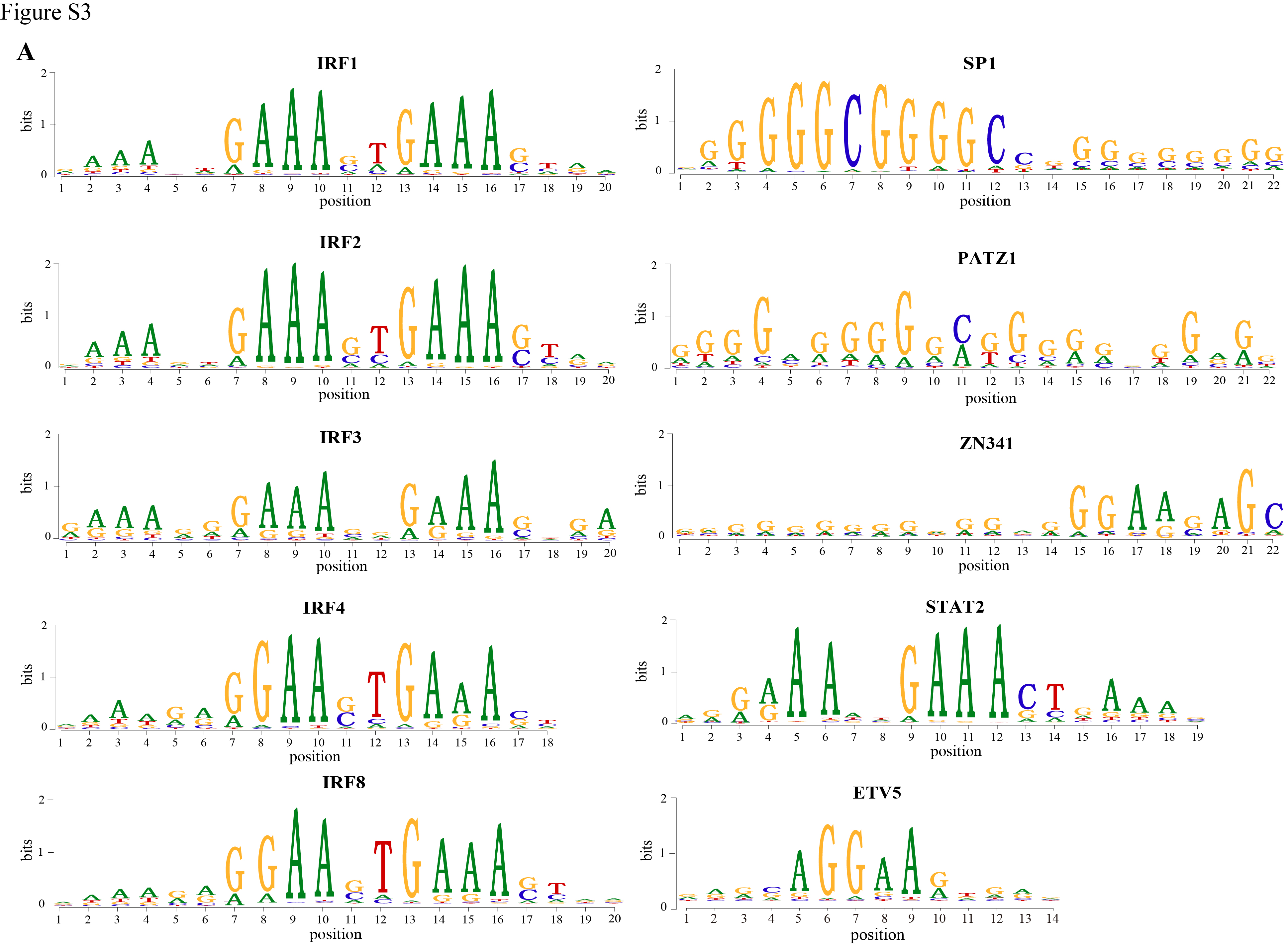

Supplement: Supplementary Figure 3 — Promoter motif analysis. Promoter motif analysis identifies significantenrichment of the IRF motif (i.e., the IRF1 binding site) and the othermotifs in each cluster (13 clusters in SY18 and 15 clusters in HuB20). Bydefault, two hundred bases upstream and fifty bases downstream of thetranscriptional start sites (TSS) sequences were analyzed in MEME (Evalue<10). [file Image_3.jpg]

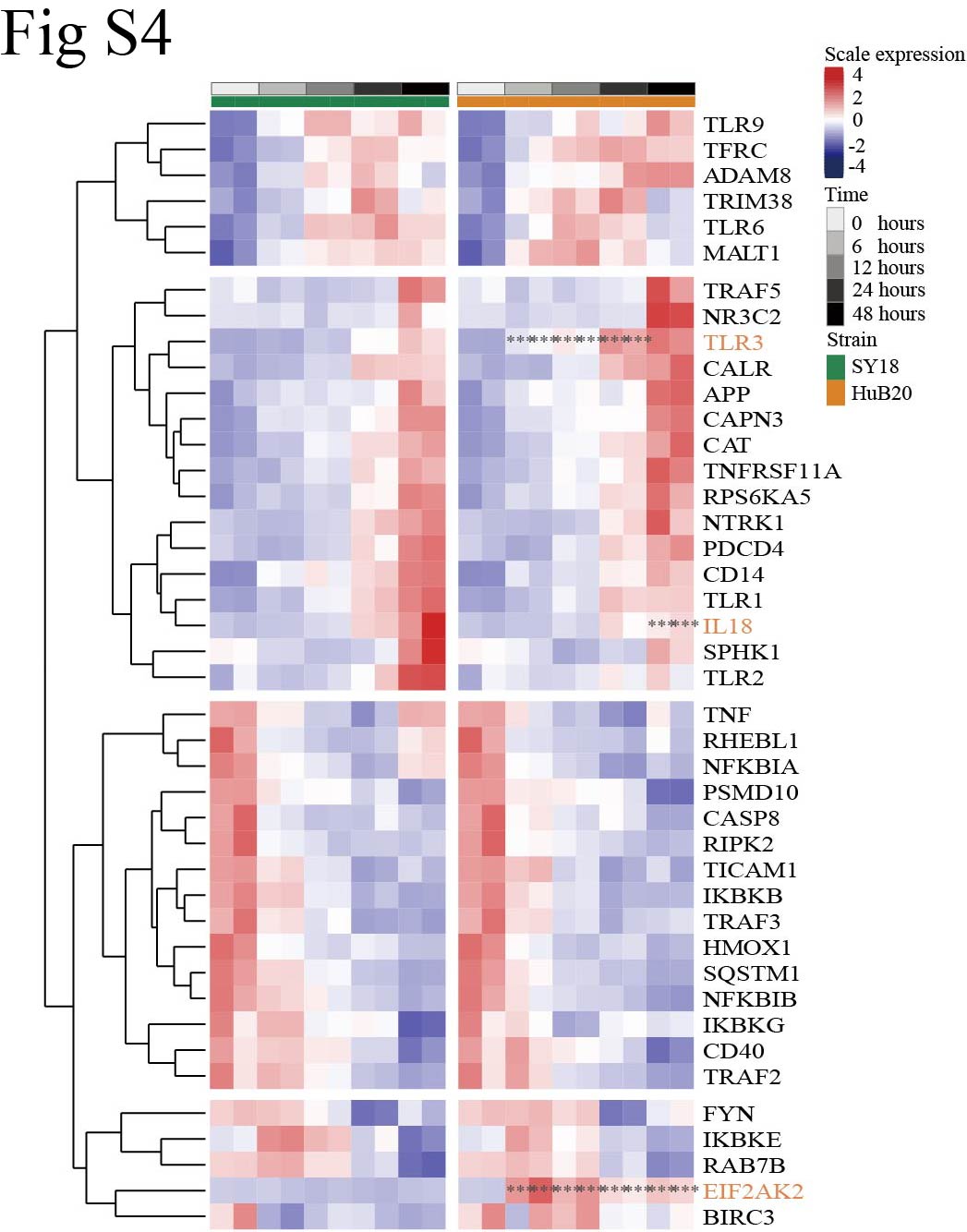

Supplement: Supplementary Figure 4 — Expression differences of NF-kappaB pathway associated genes. (A) Heatmap showing expression levels for the NF-kappaB pathway associated genes in the ASFV SY18 and HuB20 strain. [file Image_4.jpg]

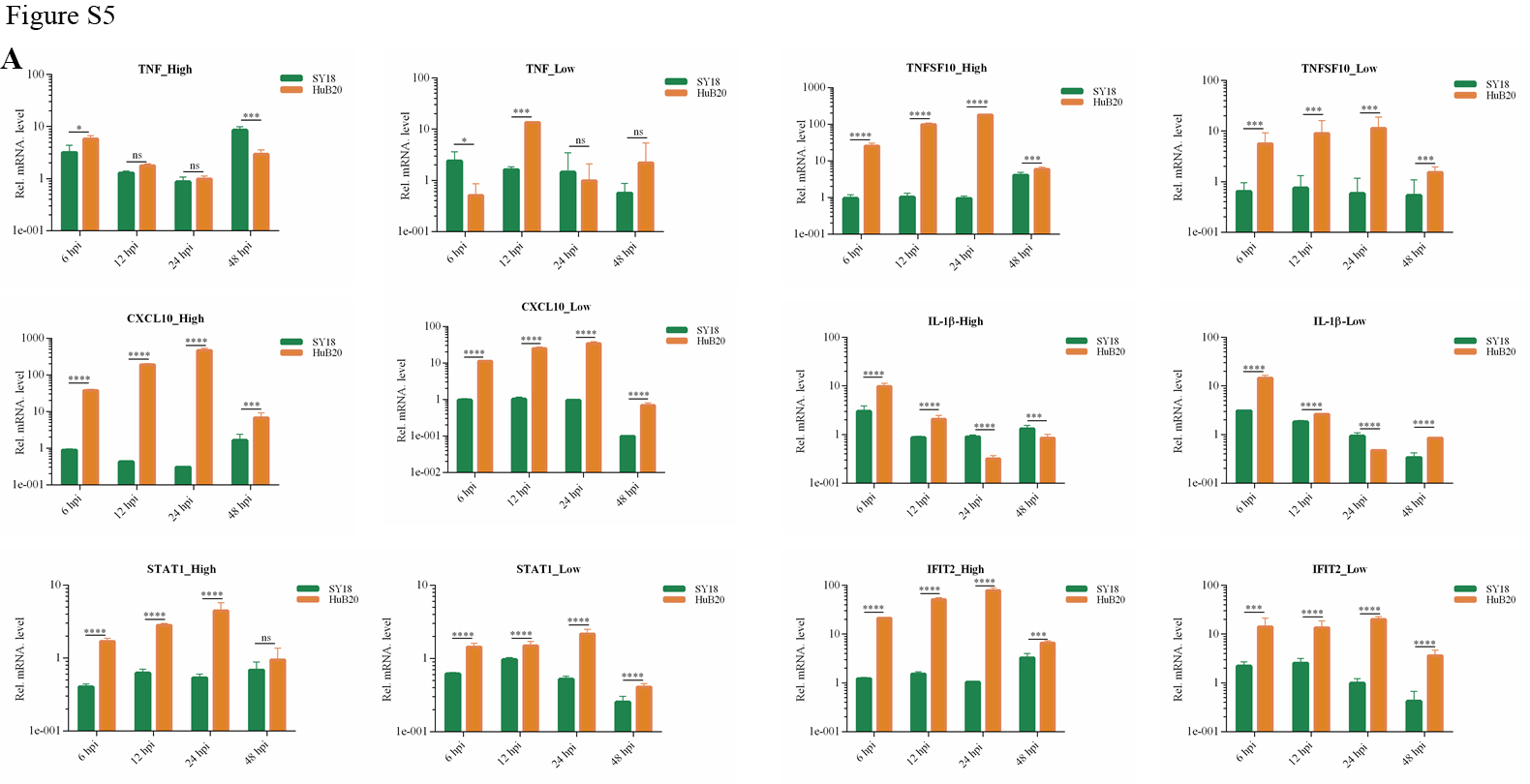

Supplement: Supplementary Figure 5 — Validation of cytokines expression by real time-PCR. PAMs were infected or mocked infected by ASFV SY18 and HuB20 strains, respectively (MOI= 1), at 6, 12, 24 and 48 hpi. Total RNA was extracted from the PAMs and subjected to RT-qPCR to quantitate TNF, CXCL10, TNSF10.IL-1β, IFIT2 and STAT1 expression (A). “High” represents PAMs with high initial TNF expression and "Low"represents PAMs with low initial TNF expression. The data were normalized using β-actin. The fold-difference was measured by the 2-∆∆Ct method.Differences were assessed using a two-sample t-test. Significance was defined at P < 0.05. *, P < 0.05, **, P < 0.01; ***, P < 0.001, ****, P < 0.0001,ns, not significant. [file Image_5.jpg]

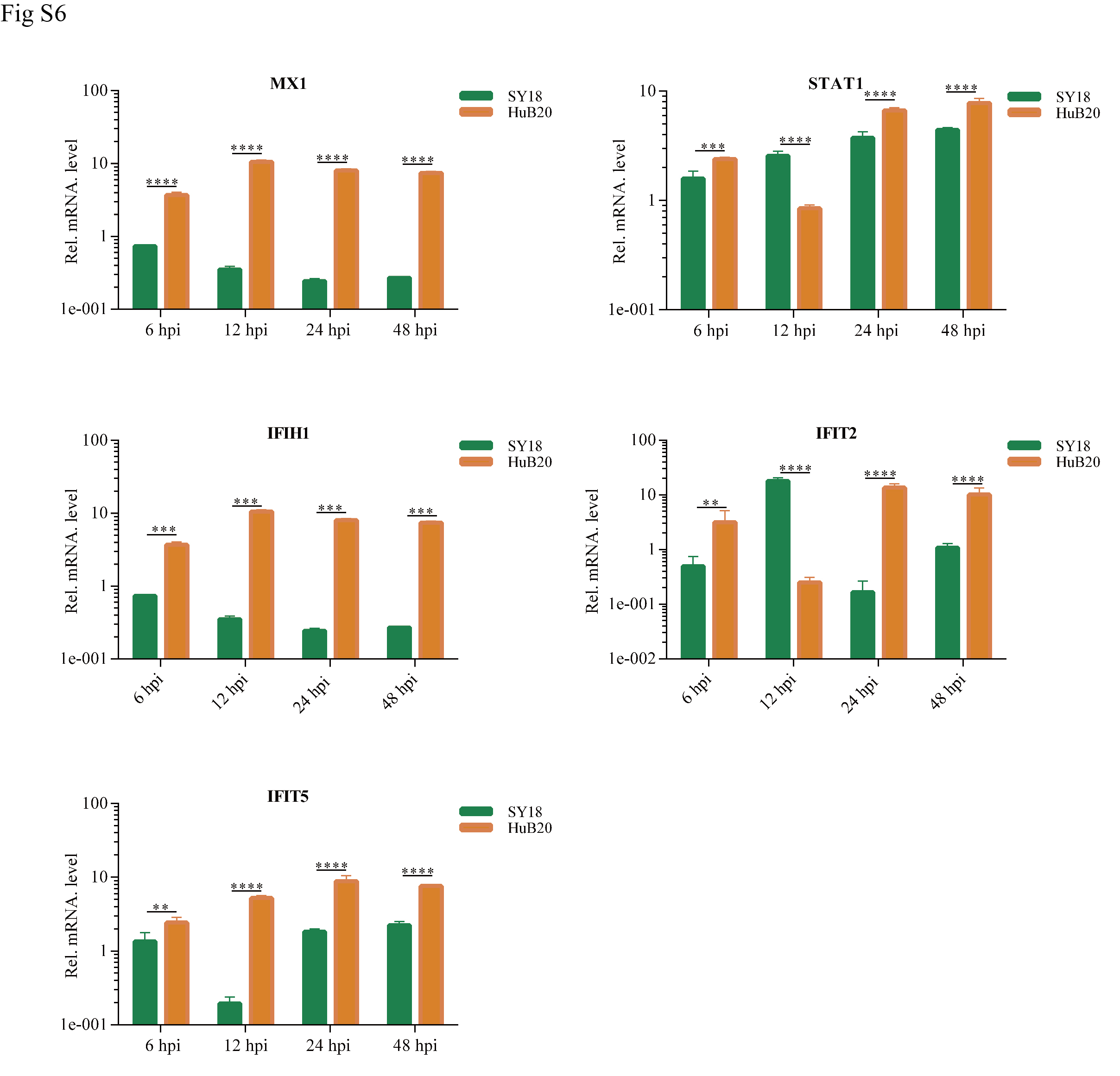

Supplement: Supplementary Figure 6 — Validation of innate immunity associated gene expression by real time-PCR. PAMs were infected or mocked infected by ASFV SY18 and HuB20 strains, respectively (MOI= 1), at 6, 12, 24 and 48 hpi. Total RNA was extracted from the PAMs and subjected to RT-qPCR to quantitate IFIH1, STAT1, MX1, IFIT2 and IFIT5 expression. The data were normalized using β-actin. The fold-difference was measured by the 2-∆∆Ct method. Differences were assessed using a two-sample t-test. Significance was defined at P < 0.05. *, P < 0.05, **, P < 0.01; ***, P <0.001, ****, P < 0.0001, ns, not significant. [file Image_6.jpg]

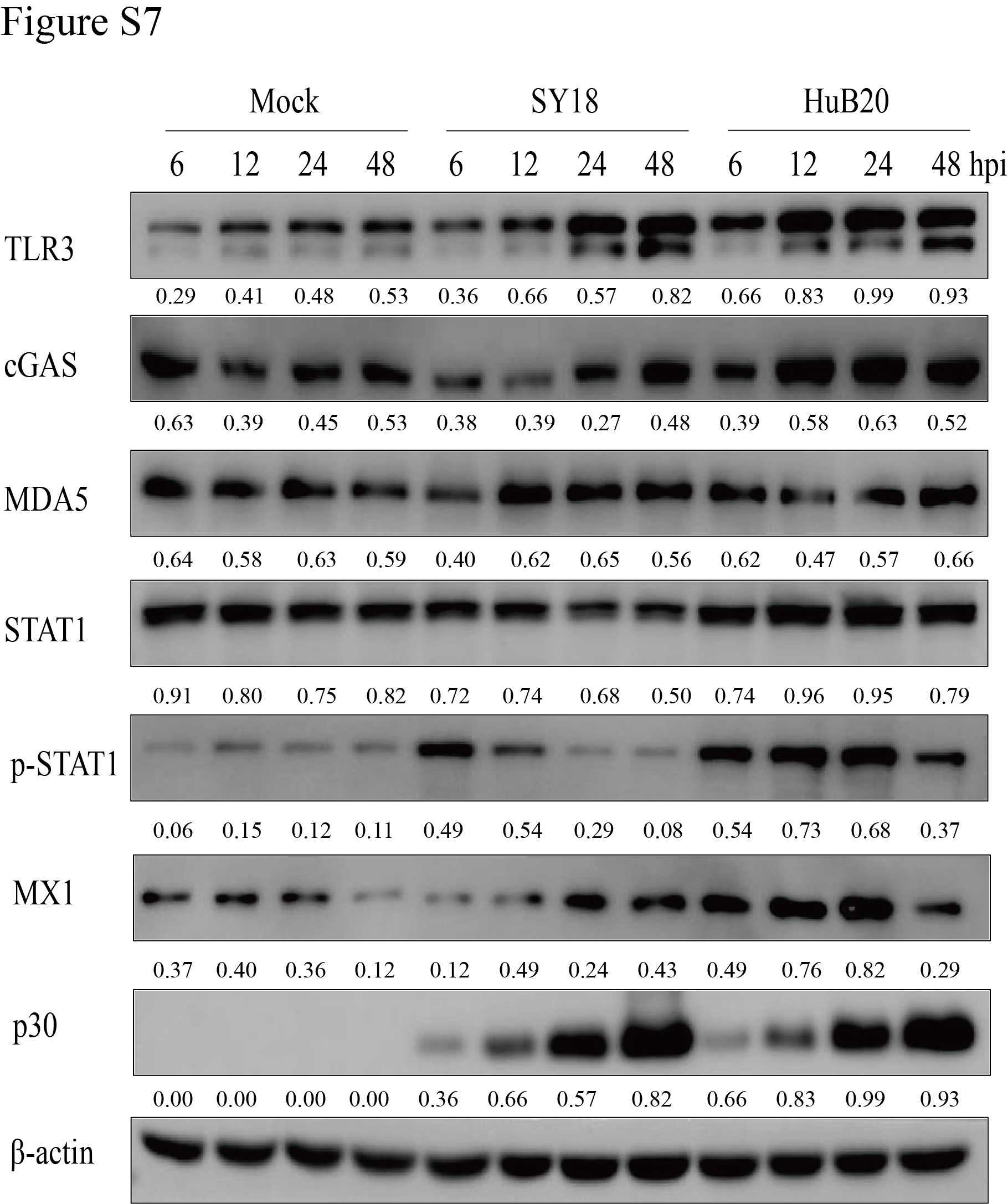

Supplement: Supplementary Figure 7 — Western blotting analysis of innate immunity associated proteins. Insertedvalues indicated relative proteins expression in comparison with b-actin. [file Image_7.jpg]

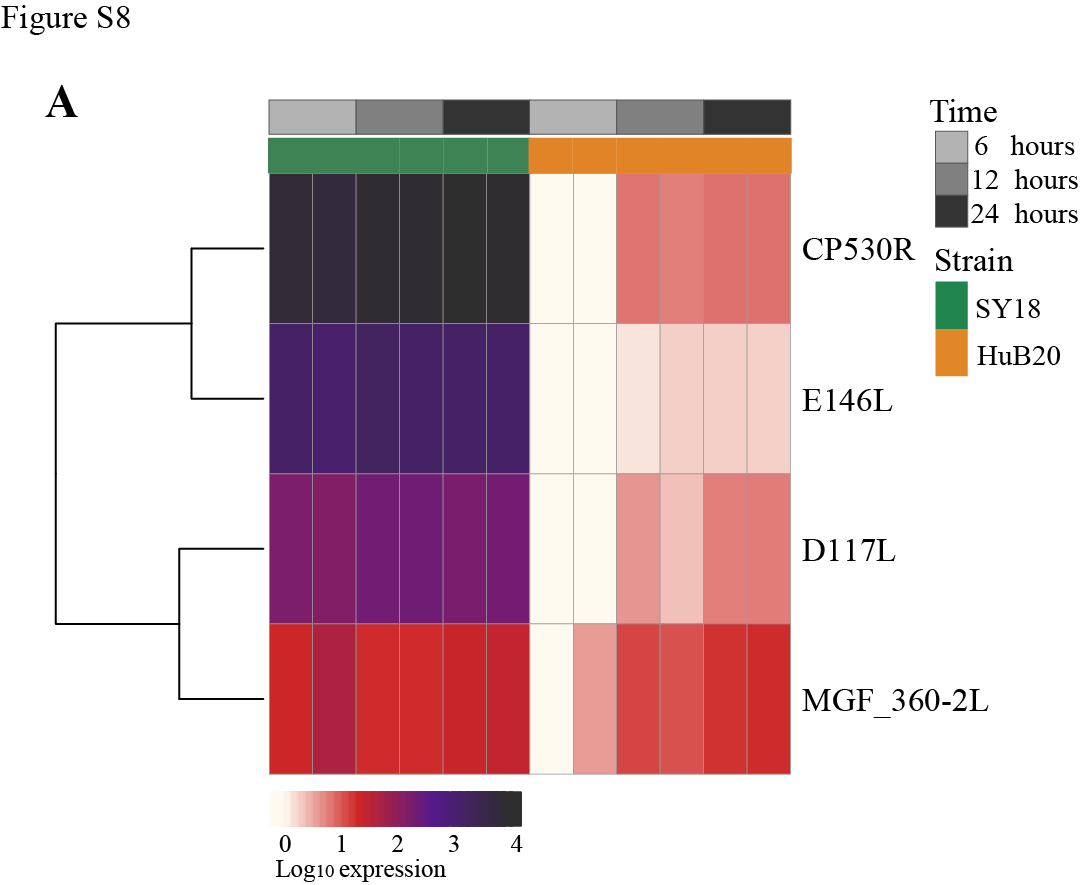

Supplement: Supplementary Figure 8 — Expression differences of selected ASFV genes. (A) Heatmap showingexpression levels for the selected virus gene CP530R, E146L, D117L andMGF_360-2L in the ASFV SY18 and HuB20 strain. [file Image_8.jpg]
